# Measuring Stratigraphic Congruence Across Trees, Higher Taxa, and Time

**DOI:** 10.1093/sysbio/syw039

**Published:** 2016-05-06

**Authors:** Anne O'Connor, Matthew A. Wills

**Affiliations:** Milner Centre for Evolution, The University of Bath, The Avenue, Claverton Down, Bath BA2 7AY, UK

## Abstract

The congruence between the order of cladistic branching and the first appearance dates of fossil lineages can be quantified using a variety of indices. Good matching is a prerequisite for the accurate time calibration of trees, while the distribution of congruence indices across large samples of cladograms has underpinned claims about temporal and taxonomic patterns of completeness in the fossil record. The most widely used stratigraphic congruence indices are the stratigraphic consistency index (SCI), the modified Manhattan stratigraphic measure (MSM*), and the gap excess ratio (GER) (plus its derivatives; the topological GER and the modified GER). Many factors are believed to variously bias these indices, with several empirical and simulation studies addressing some subset of the putative interactions. This study combines both approaches to quantify the effects (on all five indices) of eight variables reasoned to constrain the distribution of possible values (the number of taxa, tree balance, tree resolution, range of first occurrence (FO) dates, center of gravity of FO dates, the variability of FO dates, percentage of extant taxa, and percentage of taxa with no fossil record). Our empirical data set comprised 647 published animal and plant cladograms spanning the entire Phanerozoic, and for these data we also modeled the effects of mean age of FOs (as a proxy for clade age), the taxonomic rank of the clade, and the higher taxonomic group to which it belonged. The center of gravity of FO dates had not been investigated hitherto, and this was found to correlate most strongly with some measures of stratigraphic congruence in our empirical study (top-heavy clades had better congruence). The modified GER was the index least susceptible to bias. We found significant differences across higher taxa for all indices; arthropods had lower congruence and tetrapods higher congruence. Stratigraphic congruence—however measured—also varied throughout the Phanerozoic, reflecting the taxonomic composition of our sample. Notably, periods containing a high proportion of arthropods had poorer congruence overall than those with higher proportions of tetrapods. [Fossil calibration; gap excess ratio; manhattan stratigraphic metric; molecular clocks; stratigraphic congruence.]

Indices of stratigraphic congruence variously assess the difference between first occurrence (FO) dates in the fossil record and divergence times implied by the branching structure of a phylogeny (Norell andNovacek 1992; Benton and Storrs 1994; Huelsenbeck 1994; Siddall 1996; Hitchin and Benton 1997; Wills 1999; Pol and Norell 2001; Pol et al. 2004; Lelièvre et al. 2008; Wills et al. 2008; O’Connor et al. 2011; Bell and Lloyd 2015). A good fit between phylogenetic inferences and fossil dates can be regarded as mutually corroborative, and a prerequisite for using those same dates to time calibrate the tree. Developing stratigraphic congruence indices that can be interpreted straightforwardly and compared across trees is therefore important not only for paleontologists, but for all who seek to quantify evolutionary rates (Wiens 2004; Jenner et al. 2009; Clarke et al. 2011; Joyce et al. 2013; Legg et al. 2013; Sansom and Wills 2013; Wheeler et al. 2013). The most obvious application is in the use of fossils to calibrate molecular clocks (Lloyd et al. 2008; Wills et al. 2008; Davis et al. 2010; Boyd et al. 2011; Clarke and Boyd 2015). Time-calibrated trees can further reveal shifts in evolutionary rates (Bapst 2013, 2014; Puttick et al. 2014) and modes (Hunt et al. 2015) and be used to test hypotheses about the drivers of turnover. A sound understanding of phylogeny is also increasingly important for quantifying macroevolutionary patterns and trends (Steeman et al. 2010; Tanja and Folmer 2013), including the selectivity of extinction (Purvis et al. 2011; Hardy et al. 2012) and the correlation between traits (Betancur et al. 2015; Hsiang et al. 2015; Soul and Friedman 2015). The stratigraphic distributions of fossils can be used to inform or constrain phylogenetic hypotheses (Wagner 1995a, 1995b, 2000; Fisher 2008), with tip dating (Pyron 2011) and total evidence dating (Ronquist et al. 2012) approaches being increasingly implemented (Lee and Palci 2015; O’Reilly et al. 2015). However, the majority of cladograms are inferred from the distributions of morphological or molecular character states across taxa alone, and without reference to explicitly temporal data.

Unfortunately, none of the stratigraphic congruence indices proposed to date is entirely satisfactory, being variously influenced by tree balance or shape (pectinate or symmetrical), tree size (number of terminals), and the distribution of FO dates through time, among other variables. This article therefore addresses five related questions concerning the five most widely cited stratigraphic congruence indices: the Modified Manhattan Stratigraphic Metric (MSM${}^{*}$ of Pol and Norell 2001), the Stratigraphic Consistency Index Huelsenbeck 1994), the Gap Excess Ratio (GER of Wills 1999), and the topological GER and modified GER (GERt and GER${}^{*}$ of Wills et al. 2008).

1. Is it possible to compare congruence indices for alternative trees comprising the same terminals (i.e., the same FO dates and the same taxon set but different inferred relationships)? This is essential if indices are to be used as ancillary criteria for choosing between otherwise equally optimal trees (Wills 1999, 2007; Pol et al. 2004; Dyke et al. 2009; Rahman et al. 2009; Wills et al. 2009; see also Wagner and Estabrook 2015).
2. Can indices be compared across data sets (different sets of taxa and different distributions of FO dates)?
3. What is the influence of 11 factors reasoned or purported to bias these indices of stratigraphic congruence? We also consider how these factors relate to different models of diversification, and therefore how different patterns of cladogenesis might affect indices.
4. Are reported patterns of stratigraphic consistency through geological time (e.g., Benton et al. 2000; Wills 2007) and across higher taxa (e.g., Wills 2001) robust when these biases are factored out? Studies of congruence for trees binned into Phanerozoic eras (Benton et al. 2000) revealed no significant differences; a result very widely cited as demonstrating uniformity in the quality of the fossil record at gross levels of taxonomic and stratigraphic resolution (Lloyd et al. 2008; Guinot et al. 2012; Brocklehurst and Frobisch 2014; Smith et al. 2014). Finer subdivision and reanalysis of these data (Wills 2007; O’Connor et al. 2011) yielded a more complex pattern of congruence, being higher in the Mesozoic than the Paleozoic or Cenozoic. Studies of congruence across higher taxonomic groups (Benton and Simms1995; Benton and Hitchin 1996; Benton 2001) all concur that congruence for trees of arthropods is significantly inferior to that for most vertebrate and many other invertebrate groups (Wills 1998).
5. Which indices have the widest utility

We address these questions in two ways: firstly using simulations for several contrived cases, and secondly using a large empirical data set of 647 published cladograms.

**Figure 1.**
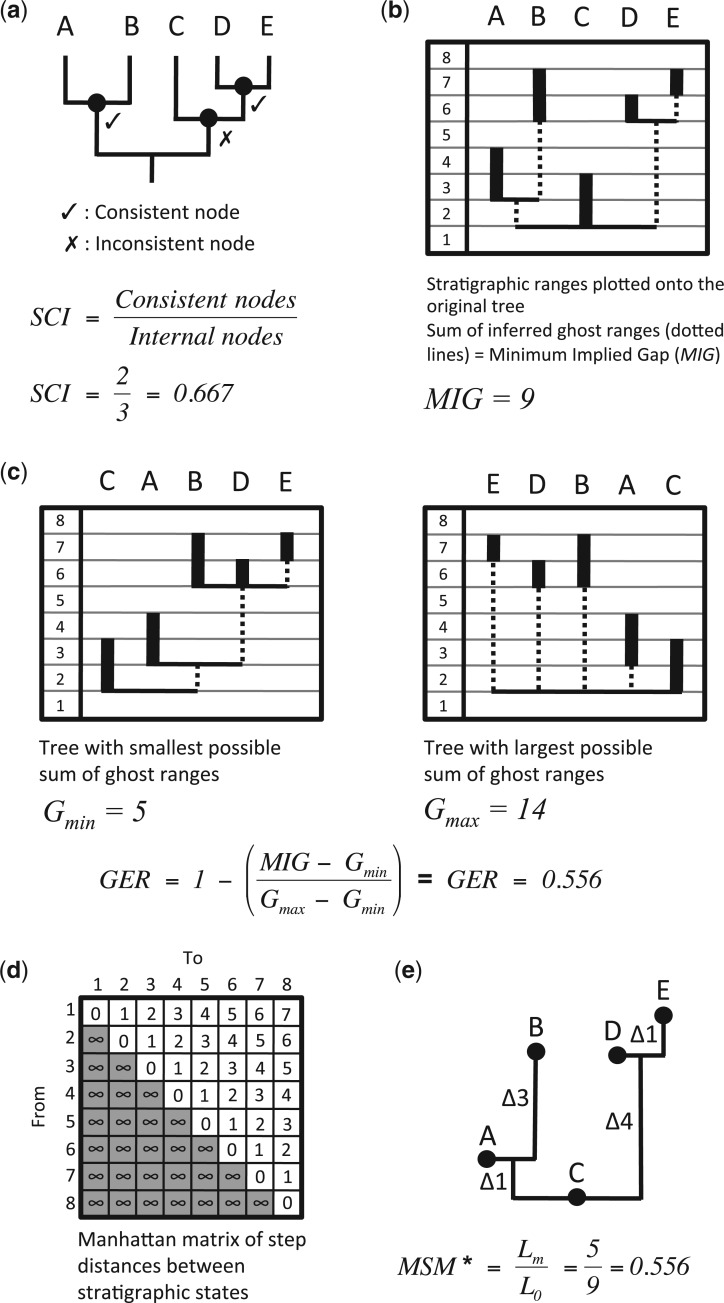
Calculation of stratigraphic indices. a) Cladogram for five taxa (A–E) with internal nodes marked as black circles. b) Observed stratigraphic ranges for taxa A–E indicated as vertical black bars through stratigraphic intervals 1–8. Cladistically implied ghost ranges (using the tree from a) are indicated by dotted lines, and the sum of the lengths of these ghost ranges (in number of intervals) gives the minimum implied gap (MIG). (a and b) The SCI is given simply as the fraction of internal cladogram nodes that have sister nodes or terminals as old or older. c) Calculating the GER. The MIG is the MIG (sum of cladistically implied ghost ranges) observed on the actual tree (see panel “b”), and $G_{\text{min}}$ and $G_{\text{min}}$ are the sums of ghost ranges for the best and worst fits of the given set of stratigraphic ranges onto any tree topology. The $MIG_{\text{u}}$ expresses the sum of ghost ranges in terms of numbers of stratigraphic intervals of unit length, whereas the MIG is a more general term that may scale in unit intervals or in millions of years. Note that $G_{\text{min}}=L_{\text{o}}$ and $MIG=L_{\text{m}}$. d) Manhattan stratigraphic matrix of the step distances between stratigraphic states 1–8 (equivalent to an irreversible, Sankoff character). e) States from “b” and “d” optimized onto the tree in “a”, with branch length distances marked. The modified MSM (MSM*) is given by $L_{\text{m}}$$L_{\text{o}}$, where $L_{\text{m}}$ is the minimum possible length of the irreversible age character (equivalent to the steps implied between the oldest and youngest intervals; here five steps), and $L_{\text{o}}$ is the observed length of the age character (here nine steps). The values of GER and MSM* are coincidentally the same (0.556) in this example.

## Measures of Stratigraphic Congruence

*Stratigraphic consistency index.*—The SCI (Huelsenbeck 1994) assesses the congruence of each internal cladogram node, with the exception of the basal node or root (Fig. 1a, b). A node is deemed congruent if the oldest of the terminals that it supports is stratigraphically no older than the oldest of the terminals supported by its sister node. The SCI is then simply the fraction of internal cladogram nodes that are congruent, and can theoretically take any value between 0.0 (completely incongruent–all nodes inconsistent) and 1.0 (completely congruent–all nodes consistent). The SCI is unique in assessing congruence in terms of how frequently taxa appear in the fossil record in the order posited by nodal distance from the root (Siddall 1996, 1998; Wills 1999). As such, it is also the only index that is a property of a cladogram rather than of a particular phylogeny within a cladogram. All phylogenies consistent with a given cladogram will have the same SCI, but may differ in terms of the other indices discussed here. These other indices directly or indirectly assess congruence using the extent of phylogenetically implied gaps. Wagner and Sidor (2000) demonstrated that the SCI tends to 0.5 as preservation rates decline, but increases as preservation rates improve. They also noted that the SCI tended to 0.5 as preservation rates declined, but increased as preservation rates improved. They also noted that the SCI tended to 0.5 as random tree error was introduced into their simulations.

*GER and derivatives*.—These indices variously utilize the concept of ghost ranges: gaps in sampling implied by a phylogeny and subtended between inferred sister taxa (Fig.1b). Sister groups (whether two terminals, two clades, or a combination of these) derive from a hypothetical ancestor. As such, the two daughter lineages must have originated at the same time, but this is seldom recorded in the fossil record; implying a gap or ghost range. Ghost ranges are equivalent to the “stratigraphic debt” of Fisher (2008), although the concept has a much older inception (e.g., Shaw 1964). For the GER (Wills 1999), ghost ranges summed across the tree (MIG) are scaled relative to the theoretical minimum ($G_{\text{min}})$ and maximum ($G_{\text{max}}$) sum of ghost ranges on *any* topology (Fig. 1b, e). GER values range from 0.0 (maximum incongruence) to 1.0 (maximum congruence). Wagner (2000) demonstrated that the expected number of gaps posited by a tree with N taxa should increase as the intensity of sampling decreases. The consistency of sampling, model of cladogenesis, and taxonomic practice are also influential. While these effects have been explored elsewhere (e.g., Wagner and Sidor 2000), their influence upon the minimum ($G_{\text{min}})$ and maximum ($G_{\text{max}})$ ghost ranges have not been investigated. Hence, it is important to see how the GER as a whole responds to these parameters, not merely the reconstructed gaps themselves. Wills (1999) and Pol et al. (2004) demonstrated that the GER is biased by tree topology; observed values can never reach the theoretical minimum or maximum on a balanced tree, as the *MIG* can never be equal to either $G_{\text{min}}$ or $G_{\text{max}}$. The GER is also comparable to the retention index (*sensu* Farris 1989) of an irreversible character coding stratigraphic age, and is therefore subject to similar biases (Finarelli and Clyde 2002).

**Figure 2.**
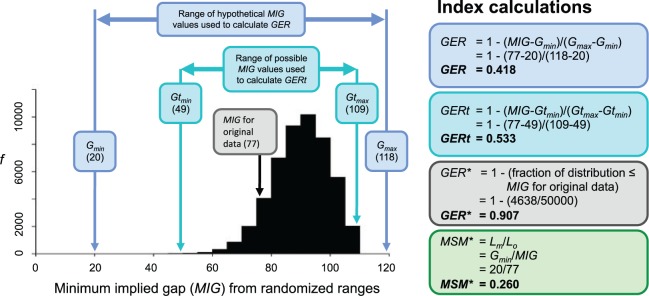
Calculation of GER, GERt, and GER* for the Mesozoic bird data of Fountaine et al. (2005). The histogram illustrates the *MIG* values derived from 50,000 randomizations of stratigraphic data over the published tree.

Wills et al. (2008) introduced two indices derived from the GER. The GERt sought to overcome the worst biases caused by differences in tree balance. This operates by scaling the observed sum of ghost ranges between the minimum and maximum possible values on the *observed* tree topology, rather than on *any* tree topology (Fig. 2). Formally, it is given by

$$\text{GERt}=1-\frac{MIG_{\text{u}}-Gt_{\text{min}}}{Gt_{\text{max}}-Gt_{\text{min}}},$$

where $MIG_{\text{u}}$ is the sum of ghost ranges for stratigraphic intervals of unit length, and $Gt_{\text{max}}$ and $Gt_{\text{min}}$ are the maximum and minimum possible values of $MIG_{\text{u}}$ on the given topology. Practically, $Gt_{\text{min}}$ and $Gt_{\text{max}}$ have been estimated by permuting the assignment of range data over the tree, such that the bounds are likely to depend upon the number of replicates. As noted by Wills et al. (2008), there are many more incongruent distributions than congruent ones, analogous to the asymmetric distribution of tree lengths for randomly generated trees. In this context, the asymmetric distribution of possible $MIG_{\text{u}}$ values means that $Gt_{\text{max}}$ is likely to more closely approximate to $G_{\text{max}}$ than $Gt_{\text{min}}$ will approximate to $G_{\text{min}}$. This rescaling typically results in values of GERt higher than the corresponding GER. To overcome the arbitrary effects of the number of replicates, a modified GER (GER*) was proposed (Wills et al. 2008). This is estimated from the fraction of the area under a curve of permuted values corresponding to a $MIG_{\text{u}}$ value greater than the observed value, and is much less sensitive to the number of permutations used (Fig. 2). The GERt and GER* have not yet been explicitly tested for biases.

*Modified Manhattan stratigraphic measure*.—The MSM* (Siddall 1998; Pol and Norell 2001) can be derived by parsimoniously optimizing an irreversible Sankoff character (Fig. 1d) coding the first stratigraphic occurrences of all terminals across a given tree (Fig. 1e). The MSM* attains a value of 1.0 when the Sankoff character is optimized with the minimum possible steps (the best possible fit), and tends toward 0.0 as the number of observed steps increases (although a value of 0.0 is never attained). MSM* is also equivalent to $G_{\min}/MIG$. A number of studies have shown that the MSM* is biased by tree size (Siddall 1998; Pol et al. 2004; Lelièvre et al. 2008), and tree shape (Pol et al. 2004; c.f. Siddall 1998). Lelièvre et al. (2008) also noted that the MSM* is analogous to the consistency index (Kluge and Farris 1969), and is therefore subject to the same biases.

As with all congruence indices utilizing inferred ghost ranges between sister taxa, the MSM*, GER, and its derivatives assume a bifurcating model of cladogenesis and the absence of any sampled ancestors (the “metataxon” definition of ancestry *sensu* Donoghue 1985; Archibald 1994). Similar assumptions underpin some other approaches that attempt to time calibrate morphological trees (Laurin 2004; Ruta et al. 2006; Brusatte et al. 2008; Bell and Lloyd 2015) and these may be reasonable where terminals have apomorphies that distinguish them from internal nodes (often the case with genera and higher taxa; but see Foote 1996; Polly 1997; Bapst 2014). We return to this issue in the discussion.

*Desirable properties of indices*.—As noted above, the SCI, GER, GERt, and MSM* all theoretically scale between 0.0 (least congruent) and 1.0 (most congruent), although these extremes are not always achievable for all indices on all trees. It is also intuitively desirable that indices should have distributions centered on 0.5 for randomly assigned stratigraphic data, and that such distributions should not be strongly skewed. Indices should also be insensitive to parameters that differ between trees: both equally optimal trees from the same data set (e.g., tree balance) and trees from different data sets (e.g., tree size and the distribution of FO dates). The GER* differs from the other indices because it is derived with reference to the distribution of *MIG* values for randomized data, and is therefore analogous to a P value (albeit scaled inversely, such that GER* values also vary from a maximum of 1.00 to a minimum of zero).

## Materials and Methods

### Simulations

Previous studies have investigated the effects of one or more potentially conflating factors upon the expected distribution of one or more stratigraphic congruence indices (e.g., Siddall 1996; Pol et al. 2004; O’Connor et al. 2011). Here, we offer a comprehensive treatment, investigating the effects of tree balance, as well as the number and distribution (i.e., regularity, clustering, and concentration toward the top or bottom of the stratigraphic range) of FO dates on all five commonly implemented indices (SCI, MSM*, GER, GERt, and GER*). Congruence indices should ideally be insensitive to variations in such parameters. We tested this here using stratigraphic data distributed randomly with respect to the branching topology. Such data should neither be especially congruent nor incongruent on average (by definition), and distributions of randomized congruence values should also be similar irrespective of the tree topology or the distribution (e.g., top- or bottom-heavy) of the stratigraphic FO dates. The simulations illustrate differences in the distributions of potential index values under differing conditions. Such differences may be sufficient to mislead inferences drawn from congruence statistics (e.g., analyses of congruence through geological time or across higher taxonomic groups).

For 64 hypothetical taxa, we coded either 4, 8, 16, 32, or 64 different FO dates, each distributed over the same range of FOs (128 intervals). These simulate aspects of different preservational regimes. Ascribing all 64 taxa to just four FO dates mimics a fossil record dominated by a small number of intensively sampled preservational Lagerstätten (e.g., the Ediacaran or the Cambrian records of soft-bodied faunas). By contrast, ascribing them to 64 distinct FO dates simulates a record with both very continuous sampling and exceptionally exact stratigraphy (e.g., having information about how high up in each formation each species is found). Ascribing taxa to 32 different FO dates might be typical for marine invertebrates, where sampling is frequently continuous and fossils can be dated in different faunal zones. Differentiating 16 different FO dates is probably more typical of the terrestrial vertebrate record, where time bins are much broader (e.g., sub-stages).

Each of these initial distributions was further distributed in three ways: regularly through time, top-heavily *sensu* Gould et al. (1977, 1987) or γ>0 *sensu* Pybus and Harvey (2000) with FOs preferentially close to the latest FO, and bottom-heavily with γ<0 and FOs preferentially close to the earliest FO. We optimized these 15 variants of FO dates onto two 64-terminal topologies; maximally balanced and maximally pectinate. We explored extremes of balance in our simulations since we have previously demonstrated that GER, SCI, and MSM* are all progressively depressed as tree balance increases (Wills et al. 1998, 2008). These extremes reflect the range of values in our empirical sample (from maximally pectinate ($I_{\text{c}}=1.00$) to nearly maximally balanced ($I_{\text{c}}=0.05$). This yielded a total of 30 different cases (precise conditions are listed in Supplementary Table S1, available on Dryad at http://dx.doi.org/10.5061/dryad.c19kb). Distributions of indices were inferred from 5000 random permutations of stratigraphic data across each tree, and GERt and GER* indices were inferred using 1000 replications.

### The Empirical Data Set

Our empirical sample comprised 647 published cladograms and their associated stratigraphic data (Supplementary Table S2, available on Dryad, and references therein). As a general rule, the size of phylogenetic data matrices (both numbers of taxa and numbers of characters) has increased with research time. Our empirical sample contains legacy matrices, deriving from the work of Benton et al. (2000), Wills (2007), and O’Connor et al. (2011). As such, our sampling may be more biased toward smaller cladograms than might be expected from an exclusive focus on the contemporary literature. The original Benton et al. (2000) data were filtered by removing trees with fewer than five FO dates (trivially small cladograms and those where FOs were concentrated in a very small number of intervals). We also removed trees that were unreferenced in Benton et al. (2000), and for which we were unable to locate the original source.O’Connor et al. (2011) expanded this data set with more recently published trees, as well as trees for hitherto little sampled groups (e.g., birds and molluscs). The resulting compilation contained trees for a wide diversity of animal and plant groups, sampled at various taxonomic levels, and spanning the entire Phanerozoic. Two principal sources were used to assemble data on the first and last occurrences of terminals: the *Paleobiology database* (http://fossilworks.org/, last accessed May 16, 2016) and *The Fossil Record* 2 (Benton 1993). These were augmented with data from the primary publications (those containing the trees) where appropriate. Stratigraphic ranges were coded to the nearest of 77 series and stages after Benton et al. (2000) and Wills (2007), from Recent (0) to Caerfai (76). All stage names were reconciled with international stratigraphic standards using the *International Stratigraphic Chart* (Remane and Ogg 2009) and *The Geologic Timescale 2012* (Gradstein et al. 2012).

All stratigraphic indices and other data set parameters were calculated using a modified version of Ghosts 2.4 (Wills 1999, 2007; Wills et al. 2008). Our general approach here and elsewhere has been to treat polytomies as hard for the purposes of calculating congruence indices. This means that all taxa within an unresolved node will subtend ghost ranges between their FOs and the FO of the oldest taxon within the polytomy. This has the practical upshot that a fully unresolved tree will yield indices of congruence at their theoretical minima. This is consistent with the view that a lack of resolution is really a lack of information. Hence, the analyses presented in the main body of the paper all resolve polytomies in the opposite sense to that implied by stratigraphic ordering. However, we have repeated all of our analyses treating all polytomies as soft and resolving them consistent with stratigraphic FO dates (see Supplementary Materials, available on Dryad). While the results of our analyses and modeling differ in some details, the overall patterns that we observe are surprisingly similar, and the conclusions that we draw remain unaltered.

### Independent Variables

We included parameters that had been investigated hitherto (justified either logically or empirically), to which were added a number of other variables that vary substantially across empirical data sets. Variables intrinsic to the tree topology

1. Number of taxa (number of terminals) within the tree.
2. Tree balance was calculated using Colless’ index of imbalance ($I_{\text{c}}$: Colless 1982), scaled such that a value of 0.0 signifies a maximally balanced tree and a value of 1.0 signifies a completely pectinate tree. Kirkpatrick and Slatkin (1993) and Agapow and Purvis (2002) determined that $I_{\text{c}}$ had good discriminatory power in simulations, and could be interpreted intuitively. It is also among the most widely implemented of indices (Heard 1992).
3. The percentage of resolved nodes in the tree, given simply by:
  $$r/{\left(n-2\right)}^{*}100,$$
  where r is the number of internal cladogram nodes and n is the number of terminals.
  Variables intrinsic to the nature and distribution of FO dates.
4. The mean age of FO dates of terminals in the tree was used as a proxy for its overall age. This parameter was removed from consideration when investigating the variation of indices through geological time.
5. The range of FO dates of terminals was used as a proxy for the duration of that portion of the tree relevant for calculating congruence. Several studies suggest that trees with a wider range of FO dates tend to be more stratigraphically congruent than those with a narrower range (Benton and Storrs 1994; Hitchin and Benton 1997; Benton et al. 1999; Wills 1999). All other things being equal, there is greater potential to sample fossils in the correct stratigraphic order when the range of FOs is longer, as opposed to when they are constrained within a shorter window.
6. The scaled center of gravity (CG${}_{\text{scaled}}$) of FO date. The CG on the scale of the stratigraphic interval measure (e.g., number of intervals or millions of years) was calculated using the formula of Gould et al. (1987):
  $$\text{CG}=\frac{\sum_{i=1}^{n}N_{i}t_{i}}{\sum_{i=1}^{n}N_{i}},$$
  where $N_{i}$ is the number of observed FOs in the ith interval, and $t_{i}$is the age of the ith interval. This was then rescaled between the age of the first and last occurrence to yield a scaled CG (CG${}_{\text{scaled}}$ tending between zero and one:
  $${\text{CG}}_{\text{scaled}}\text{​}=\text{​}({\text{FO}}_{\text{oldest}}\text{​}-\text{​}\mathrm{CG})/({\text{FO}}_{\text{oldest}}-{\text{FO}}_{\text{youngest}})\text{​},$$
  Where FO${}_{\text{youngest}}$ and FO${}_{\text{oldest}}$ are the youngest and oldest FO dates, respectively. CG${}_{\text{scaled}}$ indicated whether FO dates were more common near that of the oldest terminal (bottom-heavy: CG < 0.5), that of the youngest terminal (top-heavy: CG > 0.5), or neither (CG = 0.5). A CG${}_{\text{scaled}}<0.5$ is more typical of a rapidly and early radiating clade (Hughes et al. 2013), while a CG${}_{\text{scaled}}>0.5$ suggests a period of latency in the radiation of the group (e.g., in response to the evolution of a key innovation or the sudden availability of free ecospace) or a late increase in fossilization potential within multiple lineages (Wills 1998).
7. FO date variability (or gap variability). The temporal or stratigraphic spacing of FOs can be regarded as a proxy for the variability of preservation rate (rather than the preservation rate *per se*). FOs were ordered temporally, and the differences in the ages of successive FOs recorded as gaps. This is equivalent to the sequence of ghost ranges subtended between nodes on a maximally congruent, fully pectinate tree (i.e., the tree yielding $G_{\text{min}})$. FO date variability was then expressed as the standard deviation of gaps sizes divided by the range of gap sizes. Gap variability does not therefore depend on the absolute size of gaps, but rather on the constancy or regularity of gap sizes and the ratios between them.
  Variables intrinsic to the taxon sample
8. The percentage of terminals with no known fossil record. Foote and Sepkoski (1999) used this as a general proxy for the quality of the fossil record of large clades. Terminals known only from the living biota may subtend long ghost ranges between themselves and their nearest fossil relatives, thereby depressing congruence.
9. The percentage of terminals that are extant. Terminals that occur in the Recent (Wills 2007) may or may not also have a fossil record (as above).
10. Taxonomic rank of terminals (Benton et al. 1999). Six categories for the taxonomic rank of terminals were coded: species, genus, family, order, class, and phylum or above (coded 1–6). Super and sub ranks were subsumed into the rank to which they referred (e.g., subfamilies and superfamilies were all referred to families). Where trees contained terminals with a mixture of ranks, the median value was recorded. Thereafter, this variable has been treated as ordered. The taxonomic rank of terminals is correlated with clade age to the extent that higher taxa tend to have older FO dates, whereas lower-level taxa (e.g., species and genera) first occur throughout the fossil record. Higher taxa are also more likely to be monophyletic (an assumption underpinning the calculation of most congruence indices) than species and genera. In the latter case, paraphyletic taxa become increasingly more probable as the fidelity of the fossil record increases.
11. Taxonomic group. We recognized six categories (arthropods, echinoderms, fishes, molluscs, plants, and tetrapods) in an unordered variable. All of the trees in our sample fitted squarely within this system. Previous studies have shown that congruence levels vary significantly across higher taxa (Benton and Hitchin 1996, 1997; Benton et al. 1999; Wills 2001). This parameter was removed from consideration when we examined how indices varied across higher taxa.

### Statistical Analysis

All statistical analyses were carried out using R, version 3.2.2 (R Core Team 2015). Neither the GERt nor the GER* had distributions that could be induced to approximate normality with standard transformations. We therefore used generalized linear modeling (GLM; Faraway 2006) with a Gamma distribution for both the GERt and GER* and a normal distribution for the GER, MSM*, and SCI models (parameters determined empirically). Model reduction was implemented stepwise using Akaike’s information criterion (Akaike 1974). Models were initially constructed without interaction terms, but these were subsequently added. The data were modeled in three different ways (1–3), with each of the five stratigraphic indices as the dependent variable (15 GLMs in total).

1. All independent variables included. This model was used to determine the effect of all of the variables on each stratigraphic index.
2. All independent variables included, except for taxonomic group. This model was used to examine the residual stratigraphic index partitioned by taxonomic group, such that major sources of bias were removed.
3. All independent variables included, except for the mean age of FOs (used to assign a tree to 1 of the 12 geological time periods). This model was used to examine the residual trend in index by geological period; again removing major sources of bias.

To determine whether any of the observed differences through time or across taxonomic groups were significant, we implemented Kruskal–Wallis and subsequent post-hoc tests (Nemenyi–Damico–Wolfe–Dunn test;Hollander and Wolfe 1999).

In addition to the GLMs, we used a random forests approach to rank the most significant and influential independent variables (Breiman 2001). This used a large number (forest) of bootstrapped binary decision trees to determine the relative importance of each predictor variable upon the response (the congruence index).

## Results

### Simulations

**Figure 3.**
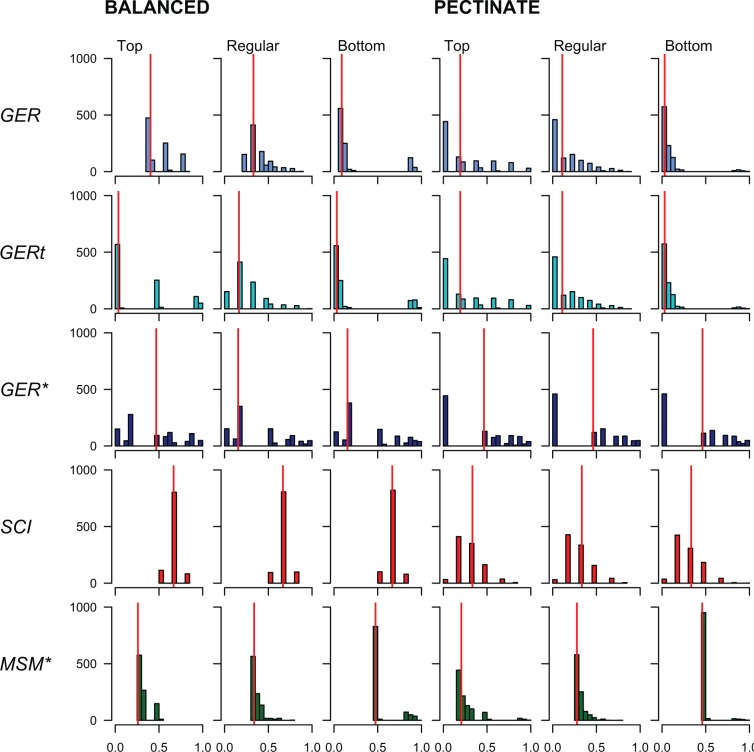
The distributions of all five indices of stratigraphic congruence (GER, GERt, GER*, SCI, and MSM*) are influenced by tree balance (balanced or pectinate) and the distribution of FO dates: whether regularly and equally spaced (regular), clustered toward the oldest FO (bottom) or clustered toward the youngest FO (top). Each histogram summarizes the distribution of index values obtained from 5000 random assignments of stratigraphic range data across the terminals of a 64-taxon tree, and represents the null expectation for stratigraphically random data. The median of each distribution is indicated by a vertical line.

**Figure 4.**
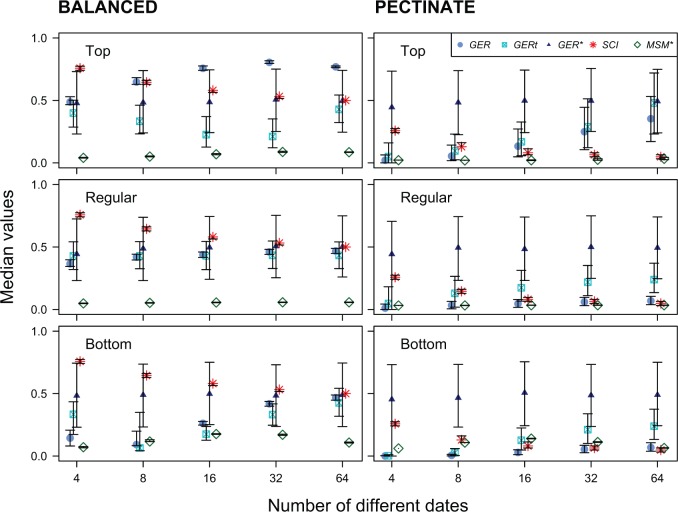
The distributions of all five indices of stratigraphic congruence (GER, GERt, GER*, SCI, and MSM*) are influenced by the number of different FO dates (4, 8, 16, 32, or 64), in addition to the distribution of those dates (regularly spaced, top- or bottom-heavy) and the balance of the tree (balanced or pectinate). Symbols indicate median indices for 5000 random assignments of stratigraphic range data across the terminals of a 64-taxon tree, while error bars denote upper (0.75) and lower (0.25) quantiles. Distributions therefore represent the null expectation for stratigraphically random data.

Of all the indices investigated, the GER* was the least influenced by any of the potentially biasing parameters, including the number of different FO dates. The median GER* value was close to 0.5 in all simulations for both top- and bottom-heavy distributions of FO dates on both balanced and pectinate trees (Fig. 3). Moreover, it was little influenced by the number of different FO dates (Fig. 4). The tendency of the GER* to a median value of 0.5 is desirable for randomly permuted data that should have congruence neither significantly better nor worse than expected.

The MSM* was somewhat more susceptible to biases than the GER*, and had much lower median values overall (Fig. 3). With regularly spaced intervals, the MSM* showed no variation with the number of FO dates (Fig. 4) on either the balanced or the pectinate tree, with a median close to 0.04 (Fig. 3). All of the other indices (GER, GERt, and SCI) showed large and significant differences over all parameters (Figs. 3 and 4). As a general observation, median values tended to be lower for a given combination of parameters on the pectinate trees than on their balanced counterparts. The GER and GERt showed a variable pattern depending upon the distribution (top- or bottom-heavy) of FO dates and tree topology.

The median SCI became progressively more depressed as the number of different FO dates increased (Fig. 4). On the balanced, 64-terminal topology, it tended toward the theoretical value of 0.50 as the number of FO dates increased up to 64 (where all values were 0.5 by definition). The SCI was entirely unaffected by the top- or bottom-heaviness of FO dates (Fig. 3). On balanced topologies, the median SCI also followed a decreasing trajectory with increasing numbers of FO dates (Fig. 4). Indeed, the median SCI on the balanced tree was greater than 0.5 wherever there were fewer FO dates than terminals; as predicted by by Wagner and Sidor (2000) and O’Connor et al. (2011). This also implies that as stratigraphic resolution increases, the SCI for balanced trees will be depressed, since all internal sister nodes comprise stratigraphically consistent and inconsistent pairs (Wagner and Sidor 2000). This would be true even for exceptionally exact stratigraphic data (e.g., ordinated beds) because the probability of two species first appearing in the same bed would still be vanishingly small. Accordingly, all median points were nearly 0.5 lower on the pectinate topologies than on their balanced counterparts, tending to a median close to 0.0 in the 64 FO date cases.

## Empirical Analyses

*Congruence indices modeled with respect to all variables*.—Each of the five congruence indices (GER, GERt, GER*, MSM*, and SCI) were modeled linearly (GLMs) with respect to all 11 of the original, potentially biasing variables, both with and without interaction terms. The minimum adequate models without interactions had between 4 (SCI) and 8 (MSM*) significant (P<0.05) parameters (Table 1), whereas the models with interactions had between 3 (SCI) and 9 (MSM*) significant parameters (Table 2).

**Table 1.** GLMs for five indices of stratigraphic congruence, excluding interactions

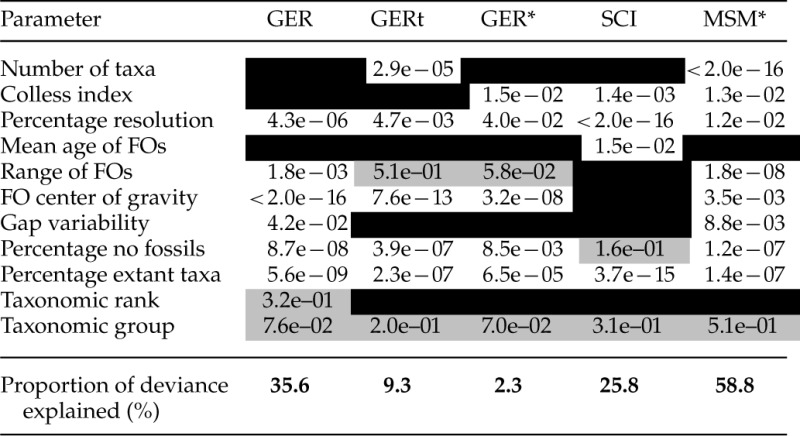

*Notes:* Summary of results from minimum adequate general linear models of each of five indices of stratigraphic congruence (GER*, GER, SCI, GERt, and MSM*) for 647 empirical trees modeled in terms of 11 independent variables, without interactions. White cell = significant parameter (P<0.05); gray cell = non-significant parameter (P>0.05) still included in minimum adequate model; black shading indicates parameter not included in final model.

Both sets of models showed broadly similar results, although some parameters that were significant on their own in the first set (without interactions) were only significant as part of an interaction in the second set. The proportion of deviance explained is a simple proxy for the extent to which each index is susceptible to sources of bias (albeit conflated with model fit). For the models without interactions, deviance was lowest for the GER* (2%), and the GERt (9%), with the highest values for the GER (36%) and MSM* (59%). Models in which polytomies were resolved stratigraphically were also closely similar (Supplementary Table S3, available on Dryad). All of the indices were strongly affected by two factors: the center of gravity of FOs (only as part of an interaction for the SCI and MSM*) and the percentage of extant taxa in the tree (only as part of an interaction for the GER*). For the GER and its derivatives, trees with a high center of gravity of FO dates (i.e., top-heavy: FO center of gravity nearest to the youngest FOs) had lower congruence than trees with a lower center of gravity (Supplementary Fig. S1, available on Dryad). All indices were negatively correlated with the percentage of extant taxa; trees with fewer extant terminals tended to have higher congruence than those with a large proportion of living representatives (Supplementary Fig. S1, available on Dryad). Percentage resolution was also significant in most cases; strongly so in the case of the SCI modeled without interactions.

Similar to the minimum adequate models, the random forest analyses including all variables highlighted the center of gravity of FO dates (i.e., whether FOs were top- or bottom-heavy) as the most influential variable affecting the GER and its derivatives (Table 3), and the third and fourth most important variable for the MSM* and SCI, respectively. Random forest analysis results where polytomies were resolved stratigraphically were closely similar (Supplementary Table S4, available on Dryad).

**Table 2.** GLMs for five indices of stratigraphic congruence, including interactions

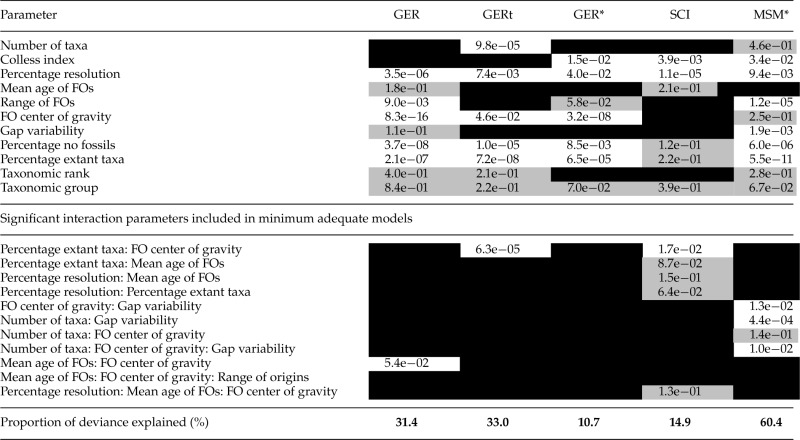

*Notes:* Summary of results from minimum adequate general linear models of each of five indices of stratigraphic congruence (GER*, GER, SCI, GERt, and MSM*) for 647 empirical trees modeled in terms of 11 independent variables, with interaction terms. White cell = significant parameter (P<0.05); gray cell = non-significant parameter (P>0.05) still included in minimum adequate model; black shading indicates parameter not included in final model. The proportion of deviance explained in each case is a proxy for the extent to which each index is susceptible to sources of bias. Hence, the relatively high proportion of deviance for the MSM* implies that the index is the least readily comparable across trees.

**Table 3.** Results of random forest analyses for five indices of congruence

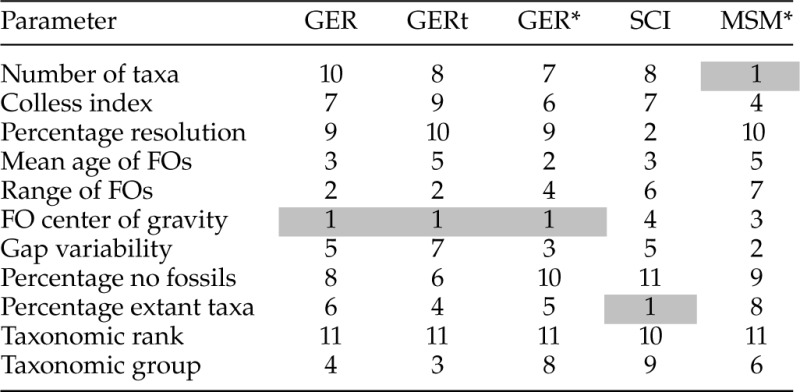

*Notes:* Independent variables are ranked in importance from 1 (highest importance) to 10 (lowest importance).

**Figure 5.**
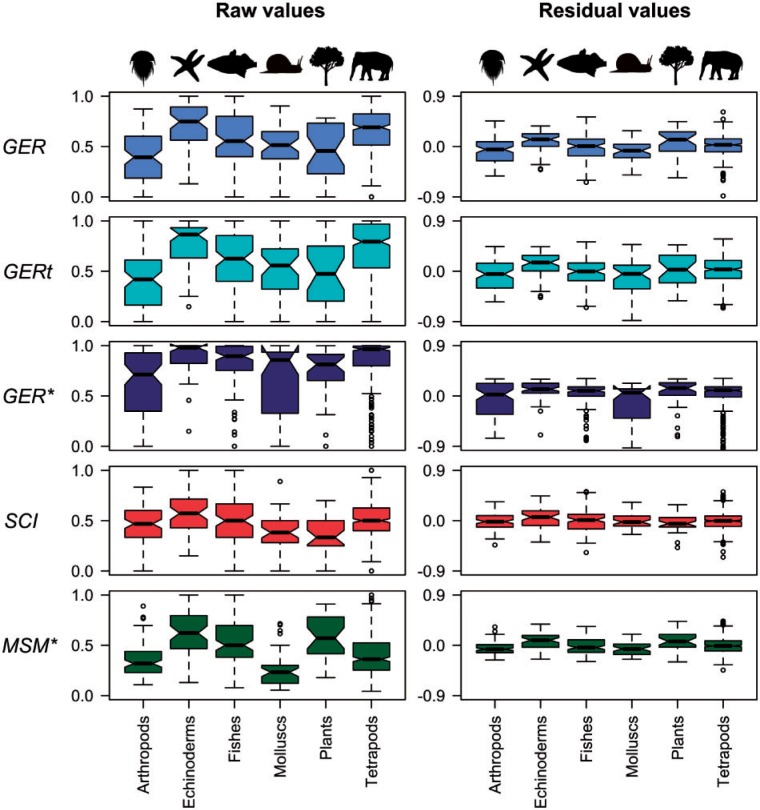
Median indices of stratigraphic congruence (GER, GERt, GER*, SCI, and MSM*) and their residuals (from minimum adequate models) vary significantly across higher taxa. Median values are indicated by black horizontal bars, shaded areas represent upper and lower quartiles, and dashed lines connect to the most eccentric points within 1.5 interquartile ranges of the medians. Outliers are shown as circles. Residuals are for the minimum adequate model (Supplementary Table S5, available on Dryad). All polytomies resolved in reverse stratigraphic order.

*Stratigraphic congruence across higher taxa*.—The minimum adequate models without higher taxonomic group were closely similar to those derived with the initial inclusion of all variables (as above) for all five indices (Supplementary Table S5, available on Dryad), and little influenced by the stratigraphic resolution of polytomies (Supplementary Table S6, available on Dryad). The random forest results were also closely similar. Plots of median index values for taxonomic groups (Fig. 5) demonstrated that overall, arthropods, plants and molluscs had lower median index values than tetrapods and echinoderms.

This pattern was almost identical when polytomies were resolved stratigraphically (Supplementary Fig. S2, available on Dryad). There were significant differences in all index values between groups, as shown by Kruskal–Wallis $\chi^{\text{2}}$ values (Table 4). The significant differences (as demonstrated by Nemenyi–Damico–Wolfe–Dunn post-hoc tests) were mainly those between arthropods and other taxa, echinoderms and other taxa, and between tetrapods and other taxa (Supplementary Table S7, available on Dryad). The stratigraphic resolution of polytomies had little effect upon this (Supplementary Table S8, available on Dryad). Differences between taxonomic group residuals from the minimum adequate models were less marked for all indices (Supplementary Table S9, available on Dryad).

**Figure 6.**
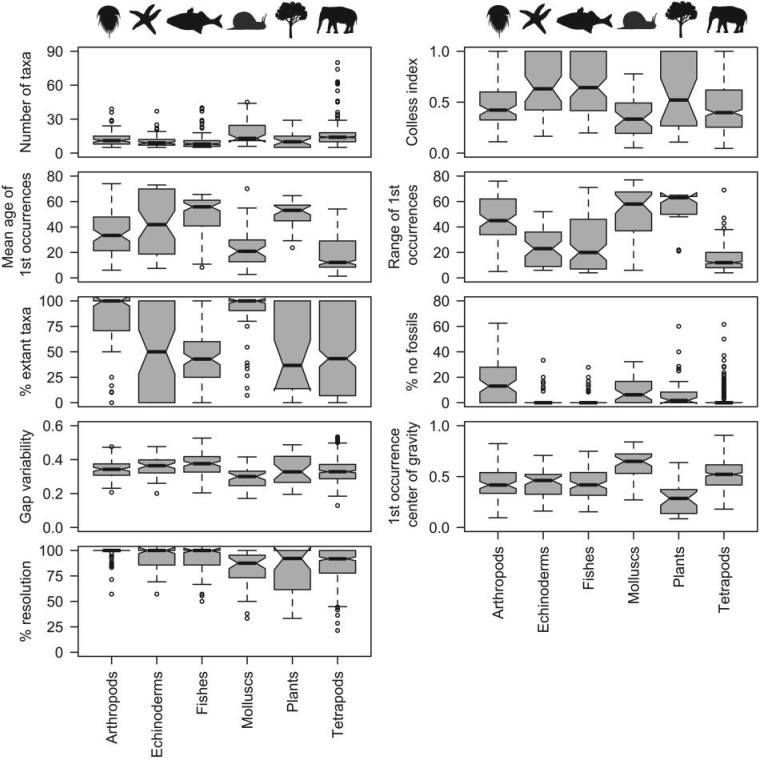
Variation in nine of the independent variables reasoned or demonstrated empirically to influence stratigraphic congruence, partitioned by higher taxonomic group. Median values are indicated by black horizontal bars, shaded areas represent upper and lower quartiles, and dashed lines connect to the most eccentric points within 1.5 interquartile ranges of the medians. Outliers are shown as circles. Mean age of FOs is measured in numbered series and stages according to Gradstein et al. (2012),from Recent (0) to Caerfai (76). Range of first occurrences is measured in numbers of series and stages on the same scale.

All of the independent variables vary significantly between taxonomic groups (Kruskal–Wallis, all values of P<3.1E−12; Fig. 6). Nemenyi–Damico–Wolfe–Dunn post-hoc tests revealed differences in gap variability, Colless’ index and number of taxa, mainly between molluscs and other groups. Both arthropods and molluscs have an extremely high percentage of extant taxa and a slightly higher percentage of taxa with no fossil record in their trees, both of which have depressive effects on stratigraphic congruence indices. If no taxa within a tree have fossil representatives, there can be no ghost ranges *MIG* will be zero by definition). However, across our sample of trees, about 6% of terminals were unknown as fossils. At relatively low frequencies, such “extant only” taxa often resolve as sister groups to terminals or clades that have a fossil record, thereby subtending ghost ranges that are frequently extensive. The center of gravity of FOs was significantly different between all groups apart from arthropods versus echinoderms, arthropods versus fishes, and echinoderms versus fishes. Unsurprisingly, mean age and range of FOs varied significantly between groups.

**Table 4.** Results from Kruskal–Wallis $\chi^{\text{2}}$ tests of median congruence across higher taxa

Parameter	$\chi^{\text{2}}$	P-value	Parameter	$\chi^{\text{2}}$	P-value
GER	94.3	<2.2e–16	GER residuals	34.7	1.8e–06
GERt	91.7	<2.2e–16	GERt residuals	25.2	1.3e–04
GER*	59.5	1.5e–11	GER* residuals	17.8	3.3e–03
SCI	43.7	2.6e–08	SCI residuals	6.9	2.3e–01
MSM*	119.2	<2.2e–16	MSM* residuals	39.7	1.7e–07

*Notes:* There are significant differences in all indices of stratigraphic congruence partitioned across higher taxonomic groups. These differences are retained (but are less significant or non-significant) when the effects of the 10 independent variables are modeled out using a minimum adequate GLM (residuals in each case). Kruskal–Wallis $\chi^{\text{2}}$ values and associated values of P, with d.f. = 5 in all cases (null states that all median values are equal in each case). For Nemenyi–Damico–Wolfe–Dunn post-hoc test results, see Supplementary Table S7, available on Dryad.

**Figure 7.**
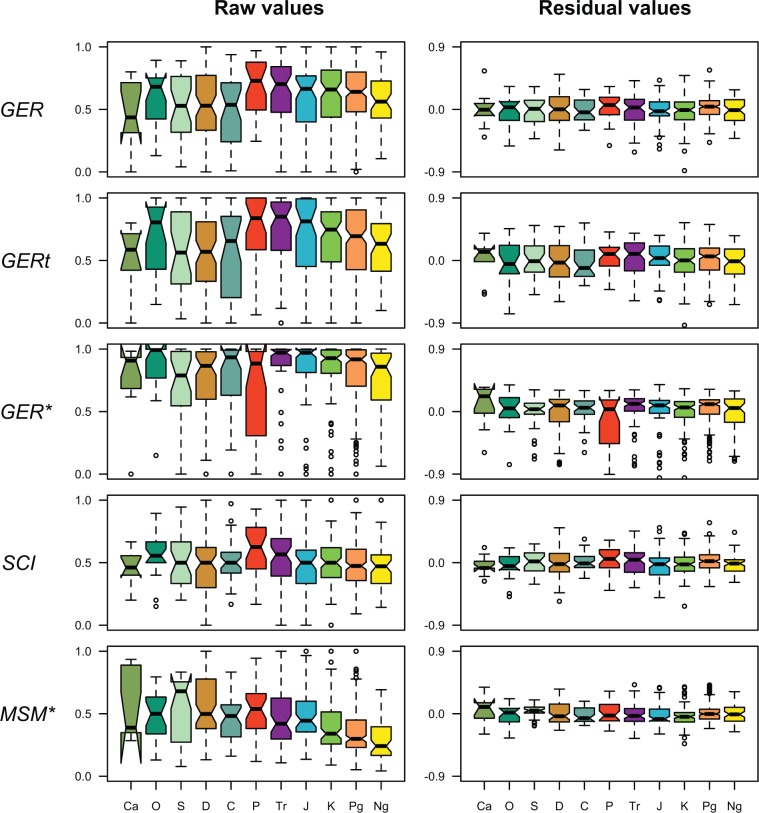
Median indices of stratigraphic congruence (GER, GERt, GER*, SCI, and MSM*) vary significantly across geological periods, while their residuals (from minimum adequate models) do not. Trees are binned according to the mean date of FO of their constituent taxa. Median values are indicated by black horizontal bars, shaded areas represent upper and lower quartiles, and dashed lines connect to the most eccentric points within 1.5 interquartile ranges of the medians. Outliers are shown as circles. All polytomies treated as hard (resolved in reverse stratigraphic order). Abbreviations: Ca, Cambrian; O, Ordovician; S, Silurian; D, Devonian; C, Carboniferous; P, Permian; Tr, Triassic; J, Jurassic; K, Cretaceous; Pg, Paleogene; Ng, Neogene.

*Stratigraphic congruence through time*.—The analysis omitting the mean age of FOs enabled us to explore patterns of residual congruence through time (Fig. 7). The minimum adequate GLMs (Supplementary Table S10, available on Dryad) and random forest results were closely similar to those from the analysis including all independent variables, and little influenced by the stratigraphic resolution of polytomies (Supplementary Fig. S3 and Supplementary Table S11, available on Dryad). Although the five indices showed patterns throughout the Phanerozoic that differed in detail, the overall trends were similar. All recorded a decline in congruence from the Permian to the Recent and an increase in congruence from the Cambrian to the Ordovician (to the Silurian in the case of the MSM*). Many periods had significantly different median MSM* values when compared with other periods (Supplementary Table S12, available on Dryad). However, none of the other cross-period comparisons were significantly different. The stratigraphic resolution of polytomies yielded similar results (Supplementary Table S13, available on Dryad), but flagged additional significant differences between the Triassic, Devonian, and Neogene for the GER* and GERt. There were no significant residual differences between any periods for any indices.

## Discussion

### Simulated Data

While all five indices are notionally scaled between zero (least congruent) and one (most congruent), only the GER* and the GERt expressed this full range of values irrespective of the tree topology or the distribution of first occurrence dates. The range of median values for the MSM* was particularly small, with median values for all simulations between 0.21 and 0.47 (a function of its scaling). In common with all previous simulations, we demonstrate a problematic sensitivity to tree balance for the SCI (Siddall 1998; Wagner and Sidor 2000; Pol et al. 2004). Our results concur with those of Pol et al. (2004), with the fully pectinate tree having a median SCI nearly 0.5 lower than the fully balanced tree for random stratigraphic data. Like Siddall (1998), we also found a decrease in median SCI as the number of different ages of FOs was increased. This is because where several dates were identical they could potentially be used to define the FOs of sister nodes, and in such cases, both nodes can be deemed congruent. For example, in the 64-taxon case with just four FO dates, there were four groups of 16 identical FO dates. When the number of FO dates was increased to 64, no two dates were identical, meaning that for any given pair of sister nodes, one must be congruent and the other incongruent. It follows that all SCI values for fully balanced trees must then be 0.50. In a similar vein, Wagner and Sidor (2000) noted that the SCI increases as sampling becomes more heterogeneous through time, mimicking the Lagerstätte effect and reducing the number of different FO dates. Similar considerations apply to gaps in the record (Wagner 2000), which become less numerous as the number of FO dates decreases.Foote et al. (1999) explained this in terms of patterns of extinction and re-radiation, leading to consequent bottlenecking and the seemingly coincident divergence of multiple groups of taxa within a relatively small number of sampled windows. If sampling becomes infrequent enough, we first encounter groups of closely related taxa simultaneously in the same intervals (Foote 2001), quite probably some time after their actual, more scattered originations. Rannala and Yang (1996) allude to a similar problem when estimating the prior probabilities of divergence times. In this way, methods that implement fossilized birth–death models (e.g., Heath et al. 2014) have many of the same strictures as attempts to quantify stratigraphic congruence.

Siddall (1996) also demonstrated that as the number of terminals increased, the distribution of possible tree topologies changed, such that the mean level of tree balance (as measured by the complement of Colless’ index of Imbalance, $I_{\text{c}})$ also increased (Colless 1982; Siddall 1996). Therefore, as the SCI is biased by tree balance and there is a relationship between the number of taxa and tree balance, there is also a theoretical negative relationship between the SCI and the number of taxa (see also Wills 1999, 2001; Pol et al. 2004; Lelièvre et al. 2008).

The MSM* was relatively insensitive to differences in tree balance in our simulations, a similar result to that reported by Pol et al. (2004). The MSM* is equivalent to the consistency index of an irreversible character (minimum possible steps divided by the observed number of steps). As such, we would expect it to behave in a similar manner to the per character ci or the ensemble CI across all characters (Kluge and Farris 1969). Huelsenbeck and Kirkpatrick (1996). and Kluge and Farris 1969). Salisbury (1999) have demonstrated that the CI is biased by tree shape, and therefore that parsimony tends to favor pectinate trees when data are noisy. The MSM* also shows relative insensitivity to the number of different FO dates in our simulations, another finding of Pol et al. (2004). Pol et al. (2004) did not test the influence of the distribution of FO dates upon any of the indices. In our simulations, both the regularly spaced and top-heavy concentrations of FO dates yielded the lowest and flattest responses as the number of different dates was varied (Fig. 4). The bottom-heavy concentration of dates showed slightly higher median MSM* values overall, with the highest median values at 16 dates in the 64-taxon case. Hence, there is some interaction between the number of different FO dates and the distribution of those dates for the MSM*.

The GER had considerable sensitivity to tree balance, but less so than the SCI. The GER was also susceptible to the number of different FO dates, especially when these were not regularly spaced, and especially on balanced trees. Ghost ranges are subtended between terminals or subclades, with the latter being dated according to their oldest constituent terminal. The pattern of subclade inclusivity therefore depends upon the branching structure of the tree. Pectinate trees have the potential to propagate ghost ranges to every node if the oldest terminal is also the most highly internested or derived (Wills 1999), whereas this propagation is more likely to be limited to a subclade in balanced trees. The larger the proportion of taxa that appear early in a clade’s history, the lower the maximum MIG (i.e., the sum of the difference between the oldest FO and all remaining FOs). This implies that when clades diversify in a manner consistent with a variety of ‘early burst’ models (Harmon et al. 2010), the GER is likely to be depressed. These include logistic diversification (Sepkoski 1979), hierarchical diversification (Brayard et al. 2009), and other models currently called “density-dependent diversification” (e.g., Rabosky and Lovette 2008; Etienne et al. 2012). A similar, depressive effect on the GER and other indices can result from declining diversity, even when sampling rates are relatively uniform.

The simulations also consider the effects of different levels of stratigraphic resolution and the completeness of the fossil record (*sensu* Schindel 1982).At one extreme, every taxon has a unique FO date, simulating a very well resolved and very complete fossil record. At the other extreme, all taxa have FOs in just four intervals. This simulates the concentration of FOs in a small number of Lagerstätten, or the situation where fossils are dated with limited accuracy. The more intermediate levels reflect what we would expect when we bin taxa into stages or perhaps zones, so that there are numerous ties despite generally good resolution.

Of all the indices investigated, the GER* was the least susceptible to the biasing factors that we investigated, while still maintaining a full range of response values (0.0–1.0 by definition). Median values for randomly permuted data were invariably very close to 0.50: a desirable property, since values of 0.5 can be interpreted as consistent with the random distribution of FO dates across the tree. In particular, the GER* was almost immune to differences in the number and temporal distribution (top- or bottom-heavy) of fossil FO dates. This means that it can be used to compare stratigraphic congruence in groups with radically different clade diversity dynamics, and with fossil records of variable fidelity both across groups and through time.

We note that the assumptions of the MSM*, GER, and its derivatives are only met where all taxa are monophyletic. This is most likely where the terminals are higher taxa (families, orders, etc., as with most of our empirical data), but its probability decreases markedly for genera and particularly species (Foote 1996). The tendency for early high morphological disparity within clades (Hughes et al. 2013) and the allied tendency for rates of character change to be greatest early in clade evolution (Oyston et al. 2015) may also result in the paraphyly of higher taxa. Rates of character change may be such that earlier and later representatives of a clade are not recognized as being constituents of the same monophyletic group, resulting in the paraphyly of the former. A variety of modifications to all ghost range-based indices could therefore be developed. The simplest would be to ignore implied ghost ranges between terminal sister species or genera (or between these and clades), assuming an ancestral relationship (ordered stratigraphically for species/genera) between these pairs. Ghost ranges would then only be inferred between “internal” sister clades in order to yield the MIG. Where morphological character data are available for the tree, a more sophisticated solution would be to require some arbitrary number of apomorphies along a terminal branch before treating it as monophyletic. Alroy (1995), Smith (2001), and Wagner (1995a) all explore how many apomorphies are likely/permissible in ancestors.

### Empirical Data

General linear models with and without interactions were broadly similar, although the former explained a higher proportion of deviance (Tables 1 and 2). The center of gravity of FOs and the percentage of extant taxa were both highly significant for all models. All indices were negatively correlated with the percentage of extant taxa in the tree (Supplementary Fig. S1, available on Dryad). This “pull of the Recent” effect (Jablonski et al. 2003) means that the stratigraphic ranges of Recent taxa (with no fossil record) tend to be extended back to their nearest fossil relatives, creating large implied ghost ranges and thereby depressing stratigraphic congruence (Wills 1999). Arthropods had the highest proportion of taxa without a fossil record (median 13.1%; Fig. 6) and the lowest congruence, while echinoderms, fishes, and tetrapods the lowest proportion and the highest congruence. Relatively poor congruence for arthropods has been noted before (Wills 2001). While more heavily mineralized, marine groups (especially trilobites and decapods) have a higher preservation potential, the majority of arthropods are terrestrial and not mineralized. Differences in preservation potential are particularly striking in Malacostraca, in which the most strongly mineralized lineages (decapods) are also the most derived, such that long ghost ranges are subtended throughout much of the tree (Wills et al. 2009). We note that the pattern of congruence indices across higher taxa is little changed when only considering trees (n=443) in which all terminals have a fossil record (Supplementary Fig. S4, available on Dryad).

The MSM* is equivalent to the consistency index of an irreversible character, which is itself biased by the number of taxa (Lelièvre et al. 2008). MSM* is negatively correlated with the number of taxa (as the number of taxa increases, MSM* decreases; Kendall’s τ=−0.48,P<2.2e−16) and positively correlated with tree balance, as measured by Colless’ index (as Colless’ index increases, moving from balanced to pectinate topologies, MSM* also increases; Kendall’s τ=0.34,P<2.2e−16). These relationships have a bearing on the relatively poor MSM* values for arthropods, molluscs, and tetrapods, since these groups have the highest median number of taxa per cladogram, along with the lowest median values for Colless’ index of imbalance (all > 0.5, indicating more balanced trees). Echinoderms, fishes, and plants have higher values (all > 0.5, more pectinate trees). Tree size was also found to be significant for the GERt and also to a lesser extent for the GER* (when interactions were included in the model).

There are many possible reasons for variation in the congruence between phylogeny and stratigraphy. Organisms vary enormously in their probability of preservation; fossilization is less likely in groups that lack a mineralized skeleton, have small body or population sizes, and in groups that have narrow geographic ranges or are restricted to particular habitats (Kidwell and Holland 2002; Smith and McGowan 2007). Arthropods, plants, and some mollusc groups have low preservation potentials and accordingly sparse fossil records (Niklas 1988; Reid et al. 1996; Friedman and Cook 2000; Brayard et al. 2010). Fossil collecting effort can also vary, with vertebrates (and dinosaurs, in particular) generating more interest than most invertebrate clades. Extremely well-studied groups may have better circumscribed FO dates and higher overall congruence as a result (Benton et al. 1999; Wills et al. 2008). Cladograms for some groups are potentially less accurate than those of others; homoplasy is higher in arthropod data matrices than in those of vertebrates (Wills 2007) and this may influence phylogenetic accuracy. Simulations demonstrate that inaccurate trees are likely to increase the summed length of ghost ranges (Fox et al. 1999; Wagner 2000) and reduce nodal congruence (Wagner and Sidor 2000) thereby depressing the SCI. Wagner (2000) and Hoyal-Cuthill et al. (2010) noted that levels of inferred homoplasy are positively correlated with the number of taxa in a data matrix. Cladograms of arthropods, molluscs, and plants have higher numbers of terminals and lower congruence than the trees of most groups in our empirical sample. Tetrapod cladograms, however, have the highest median number of terminals, but also have high congruence.

Siddall (1998) observed that metrics designed to measure a particular phenomenon should be correlated with each other if they are each appropriately sensitive to that phenomenon. For our empirical sample of trees, it is reassuring to report that these relationships hold relatively strongly (Supplementary Fig. S5, available on Dryad). However, we note that the MSM* is less strongly correlated with all of the other indices (Kendall’s τ⩾0.19 and ⩽0.37;P<7.3e−13) than the other indices correlate between themselves (Kendall’s τ⩾0.36 and ⩽0.76;P=10e−20).

The variation in congruence through time was broadly similar for all five indices, and comparable to patterns seen in previous studies (Wills 2007; O’Connor et al. 2011). In general, congruence was higher throughout the Mesozoic in comparison to the Paleozoic and Cenozoic, although the MSM* did not precisely follow this pattern. The variation in congruence throughout the Phanerozoic appeared to follow the taxonomic composition of our sample (Supplementary Fig. S6, available on Dryad). In the Cambrian, 56% of trees were of arthropods (relatively low congruence) while 44% were of echinoderms (relatively high congruence). By the Ordovician, only 26% of trees were of arthropods while 70% were of echinoderms. This was reflected by an increase in all indices of congruence from the Cambrian to the Ordovician (Fig. 6). Similarly, the proportion of tetrapods (high congruence) increased from 35% to 75% from the Carboniferous to the Permian, which may account for the increase in congruence on all indices between these periods. While all indices (except the MSM*) indicate relatively high congruence from the Permian up to the Neogene, we do detect a small decline. Wills (2007) also observed this decline and attributed it, in part, to an increase in “gappiness” in the Neogene. This result is also consistent with the observation that congruence was depressed as the fraction of extant taxa in a tree increased, coupled with the unsurprising observation that the percentage of extant taxa increased significantly close to the Recent (Supplementary Fig. S7, available on Dryad). Post-hoc tests also revealed that the percentage of taxa with no fossil record is significantly higher for trees in the Neogene compared with other time periods. Gap variability does not change to a great extent through our timeframe, but it is significantly different in a number of time periods (e.g., Paleogene versus Devonian and Jurassic), as is the number of taxa (Paleogene vs. Carboniferous, Devonian, Jurassic, and Silurian; Neogene vs. Carboniferous, Devonian, Jurassic, and Silurian; Devonian vs. Cretaceous, Ordovician, and Triassic).

We also note that the center of gravity of FO dates is negatively correlated with clade age in a classic Gouldian fashion; more bottom-heavy clades in the Paleozoic and more top-heavy clades toward the Recent (Gould et al. 1987). Trees originating closer to the present typically have a shorter range of FO dates than older trees. However, Ordovician, Devonian, Permian, and Triassic trees also have lower ranges of FO dates, while Silurian trees have the largest range of all.

## Conclusions

1. Comparing trees from the same data set. None of the published indices of congruence investigated (SCI, MSM*, GER, GERt, and GER*) was entirely immune to the potential sources of bias that afflict empirical data sets. However, trees from the same data set all have the same leaf set and distribution of FO dates, such that tree balance is the only potentially biasing factor when seeking to choose between otherwise equally optimal trees from the same matrix (Huelsenbeck 1994; Wills 1999, 2007; Pol et al. 2004; Dyke et al. 2009; Rahman et al. 2009; Wills et al. 2009; see also Wagner and Estabrook 2015). Simulations show that the SCI, GER, and GERt are all depressed on more pectinate trees, while the MSM* is less sensitive by virtue of its scaling. The GER* is the least sensitive to differences in balance overall, additionally returning distributions with a median of 0.5 for randomized FO dates.
2. Comparing trees across data sets. The most significant sources of bias for all indices pertained to the number and distribution of FO dates and the sizes of trees; particularly the center of gravity of FO dates and the number of different FO dates. These parameters are all subject to marked variation across data sets, in addition to tree balance. Different models of clade diversification predict particular patterns of FO dates, with models that approximate an early burst tending to depress all indices apart from the GER* and SCI. The concentration of FOs within a small number of intervals (simulating sites of exceptional preservation or a poorly sampled fossil record) depressed the GER, GERt, and MSM, but raised the SCI. Simulations again revealed that the GER* is the index least sensitive to these biasing factors, and most suitable for cross-data set comparisons (Wills et al. 2008) and meta-analyses. Given the additional computational requirements of the GER* relative to the other indices, the GLM approach offers an alternative strategy for mitigating against the worst of these biases in large empirical studies (Benton et al. 2000; Wills 2001; 2007; O’Connor et al. 2011).
3. Utility of indices. The GER* is the most generally applicable index in the widest variety of circumstances. The general linear models in our empirical study revealed that the 11 independent variables (without interactions) accounted for just 2% of the deviance in the GER*, compared with 9% for the GERt and 26% or over for the other indices. The MSM* fared worst, with 59% of deviance attributable to the model. In more general terms, many of the factors that influence the expected distributions of stratigraphic congruence indices also impinge upon attempts to specify the prior probabilities of divergence times, and to specify the parameters of fossilized birth–death models (which underpin tip dating approaches;Footeet al. 1999; Heath et al. 2014). These factors include changes in preservation potential through time, the concentration of FO dates in a limited number of preservation Lagerstätten, and the dynamics of clade diversification (e.g., early burst vs. random walk and single selective peak models; Harmon et al. 2010).
4. Trends in congruence through time and across higher taxa largely result from differences in data set parameters. Previous empirical studies have demonstrated significant variations in stratigraphic congruence through geological time, and across higher taxa. We replicate these findings here. Specifically, we demonstrate that the GER, GERt, and SCI are highest in the Permian, Triassic, and Jurassic, while the GER* and MSM* show more complex patterns. However, when biasing factors are modeled out, the residuals show markedly less variation, and congruence through time is more uniform. Similarly, congruence is higher for echinoderms and tetrapods than other groups, contrasting with much lower values for arthropods (differences are least marked for the GER*). Again, residuals values reveal that many of these differences are a function of variations in data set parameters.

## Supplementary Material

Data available from the Dryad Digital Repository: http://dx.doi.org/10.5061/dryad.c19kb.

## Funding

This work was supported by the Leverhulme Trust [grant F/00 351/Z] and the Biotechnology and Biological Sciences Research Council [grants BB/K006754/1 and BB/K015702/1] awarded to M.A.W., and by the The John Templeton Foundation [grant 43915].
